# Safety and Efficacy of Chimeric Antigen Receptor T-Cell Therapy for Glioblastoma: A Systemic Review and Meta-Analysis

**DOI:** 10.3389/fonc.2022.851877

**Published:** 2022-05-26

**Authors:** Jong Keon Jang, Junhee Pyo, Chong Hyun Suh, Hye Sun Park, Young Kwang Chae, Kyung Won Kim

**Affiliations:** ^1^Department of Radiology and Research Institute of Radiology, Asan Medical Center, University of Ulsan College of Medicine, Seoul, South Korea; ^2^Asan Medical Center, Department of Biomedical Engineering, College of Medicine, University of Ulsan, Seoul, South Korea; ^3^Department of Imaging, Dana Farber Cancer Institute, Harvard Medical School, Boston, MA, United States; ^4^Department of Radiology, Brigham and Women's Hospital, Harvard Medical School, Boston, MA, United States; ^5^Robert H. Lurie Comprehensive Cancer Center, Department of Medicine, Feinberg School of Medicine, Northwestern University, Chicago, IL, United States

**Keywords:** chimeric antigen receptor T-cell, glioblastoma, objective response rate (ORR), overall survival (OS), adverse effect

## Abstract

**Background:**

Chimeric antigen receptor (CAR) T-cell therapy is a promising treatment option for patients with refractory hematological malignancies. However, its efficacy in glioblastoma remains unclear. Here, we performed a systematic review to summarize the safety and efficacy of CAR T-cell therapy in glioblastoma.

**Methods:**

The PubMed, EMBASE, and Cochrane databases were searched to identify articles published before June 30, 2021 describing the use of CAR T-cell therapy in glioblastoma. Information on the toxicity of CAR T-cell therapy was summarized. The pooled objective response rate (ORR) and overall survival (OS) of patients who underwent CAR T-cell therapy were estimated using a random-effects model with an inverse-variance weighting model and quantile estimation method, respectively.

**Results:**

Of 397 articles identified, eight studies including 63 patients with recurrent glioblastoma treated with various CAR T-cell regimens were included in the analysis. Six (9.5%) patients developed cytokine release syndrome (grade ≤2), and 16 (25.4%) experienced non-critical neurological events. The pooled ORR was 5.1% (95% confidence interval [CI], 0.0–10.4; *I*^2^ = 0.05%), and the pooled median OS was 8.1 months (95% CI, 6.7–9.5; *I*^2^ = 0.00%).

**Conclusion:**

Although *CAR T*-cell therapy is a relatively safe therapeutic option in patients with glioblastoma, it shows marginal efficacy, suggesting that further research is necessary for its translation into clinical practice for the treatment of recurrent glioblastoma.

## Introduction

Glioblastoma is the most common primary brain malignancy in adults, and it has a dismal prognosis (1). The current treatment consists mainly of surgery followed by concurrent chemoradiotherapy. The results of clinical trials aimed at optimizing combination therapies to improve survival (2, 3), remain unsatisfactory, and the median overall survival (OS) of patients with glioblastoma is 15–18 months (1), underscoring the urgent need to identify effective treatment options.

Immunotherapy is a major breakthrough in the treatment of advanced cancer, and it is associated with favorable survival outcomes and an acceptable safety profile (4, 5). Chimeric antigen receptor T (CAR T) lymphocytes genetically engineered to eliminate specific tumor antigens have recently gained attention as a promising immunotherapy in relapsed or refractory hematological malignancies (6). Unlike the results in hematological malignancies, CAR T-cell therapy shows limited efficacy in patients with solid tumors, and a pooled objective response rate (ORR) of 4–9% was reported in a recent meta-analysis (6, 7).

The efficacy of CAR T-cell therapy in glioblastoma patients has been investigated in several studies, and a wide range of response rates (0–100%) has been reported (8, 9). Adverse effects are relatively uncommon in glioblastoma compared with those in hematological malignancies (8, 9). Although the effects of CAR T-cell therapy on glioblastoma remain unclear, to the best of our knowledge, no attempt has been made to generate an evidence-based systematic summary of the overall efficacy and safety of CAR T-cell therapy in glioblastoma. Thus, in this study, we performed a systematic review and summarized the efficacy and safety of CAR T-cell therapy in glioblastoma to optimize its application. In addition, evidence-based perspectives on challenging issues were extracted from the included studies.

## Materials and Methods

This study was conducted and reported based on the Preferred Reporting Items for Systematic Reviews and Meta-Analysis (PRISMA) guidelines (10).

### Literature Search and Quality Assessment

A comprehensive literature search of MEDLINE, EMBASE, and Cochrane databases was performed to identify relevant studies analyzing the use of CAR T-cell therapy in the treatment of brain tumors. The search terms used were “CAR T-cell therapy”, “immunotherapy”, and “brain neoplasm”. The details of the literature search are provided in **Supplementary Table 1**. The search included studies published until June 30, 2021, without setting a starting date, and was limited to articles published in the English language.

Study quality was assessed using the Newcastle Ottawa Scale (NOS) (11). Each study was evaluated on three categories: 1) the proper selection of the study population, 2) the comparability of the study groups, and 3) the ascertainment of the exposure or outcome of interest. Two reviewers independently assessed the quality of each study. Studies with a score ≥7 (out of a maximum of 9 points) were considered high quality studies. Studies that were ambiguous or generated differences in opinion between the two reviewers were re-evaluated at a consensus meeting that included a third reviewer.

### Study Selection

After eliminating studies that overlapped between databases, the eligibility of each article was evaluated according to predefined inclusion and exclusion criteria. The study inclusion criteria were as follows: 1) inclusion of patients who underwent CAR T-cell therapy for glioblastoma, and 2) assessment of clinical response following CAR T-cell therapy. The exclusion criteria were as follows: 1) studies not related to the topic of interest of this study; 2) *in vitro* or animal studies; and 3) review articles, editorials, letters, conference abstracts, and proceedings.

Articles were first screened by reviewing titles and abstracts, and studies were eliminated according to the exclusion criteria. The full texts of the remaining articles were then reviewed to determine their eligibility. The two reviewers selected studies independently in two sequential review sessions.

### Data Extraction

The following data were retrieved from each study: 1) study details, including authors, year of publication, and study type and design; 2) study patient characteristics, including age, sex, and the number of patients; 3) glioblastoma characteristics, including type (recurrent or naïve glioblastoma), previous treatment, and genetic profile; 4) CAR T-cell therapy details, including target antigen, co-stimulators, pre-treatment such as lymph depletion or tumor resection, T-cell dose, and drug delivery methods; 5) study outcome, including objective response based on best overall response (BOR), OS, and CAR T-cell persistence; and 6) adverse reaction details, including maximum tolerated dose, number of patients with CRS and/or CAR T-cell-related encephalopathy syndrome (CRES) (12) or other neurologic events and their grades along with consequences.

### Statistical Analysis

Six studies with three or more patients (8, 13–17) were included in the meta-analysis. The pooled ORR based on the BOR was obtained using a random-effects model with an inverse-variance weighting model (18). The pooled median OS was calculated using the quantile estimation method (19). The heterogeneity of results among the included studies was examined using Cochrane’s Q-test and Higgins’ *I^2^* statistic. Publication bias was visually assessed in a funnel plot and confirmed by Begg’s test and Egger’s test (20). In cases of publication bias, trim-and-fill adjustment was performed to calculate the adjusted pooled ORR and pooled median OS. All *P*-values were two-sided, and a *P*-value < 0.05 was considered statistically significant. Statistical analyses were performed using the “meta”, “metamedian”, and “metafor” packages in the R version 3.6.3 (R foundation for Statistical Computing, Vienna, Austria).

### Qualitative Review

To explore the clinically relevant issues, we addressed the following questions:

1. Is generation of CAR technology associated with safety and persistence?
2. Is locoregional administration of CAR T-cells associated with clinical outcome?
3. Is CRS or CRES development significant in the treatment of glioblastoma with CAR T-cell therapy?
4. What is the common mechanism of recurrence or progression after CAR T-cell therapy?
5. Are conventional brain magnetic resonance imaging (MRI) findings accurate for evaluating the treatment response*?*

## Results

### Literature Search and Quality Assessment

**Figure 1:**
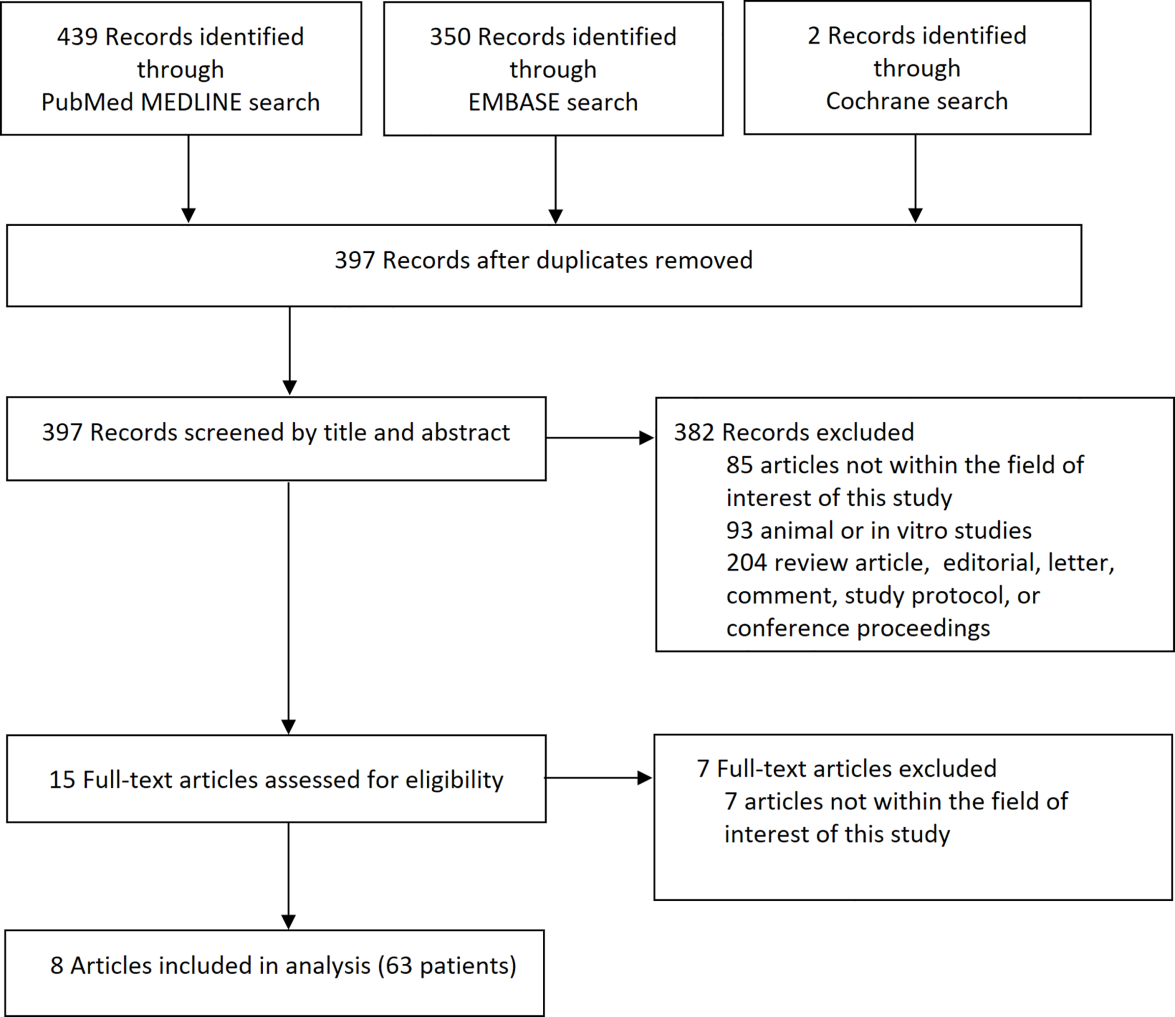
Flow diagram of the article selection process. An article may have been excluded for multiple reasons, but only 1 major reason per article is presented.

The article screening and selection process is presented in **Figure 1**. Of 397 articles identified after removal of overlapping studies between databases, 382 articles were excluded after screening titles and abstracts. The remaining 15 articles were evaluated for eligibility according to full text, and eight articles with 63 patients were finally included.

The NOS scores assigned to each study ranged from 4 to 6 points, with a median value of 5 points (**Supplementary Table 2**). With respect to the selection of cohorts, one study scored 3 points and seven scored 2 points. Regarding the comparability of cohorts, none of the studies scored any points. Regarding outcomes, six studies scored 3 points and two studies scored 2 points. None of the studies received a NOS score ≥ 7.

### Study Characteristics

The characteristics of the eight studies are shown in **Table 1**. Five were phase 1 studies (8, 9, 14, 15, 17), whereas the nature of others was not mentioned. All studies (n = 63) (8, 9, 13–17, 21) used CAR T-cell therapy for recurrent glioblastoma, and 59 patients in five studies (8, 9, 14–17) had received previous treatments consisting of surgical resection, concurrent chemoradiotherapy, and chemotherapy using temozolomide. There were five glioblastomas containing a O^6^-methylguanine-DNA methyltransferase (MGMT) promoter hypermethylation and 16 glioblastomas had no evidence of MGMT promoter hypermethylation. Patient age ranged from 30 to 77 years.

**Table 1:** Characteristics of the included studies.

Study	Study phase	No. of patients (% male)	Type of tumor	Previous treatment	Genetic profile	Pre-treatment	Target	Drug delivery	T-cell dose,/m^2^
Brown et al. (13)	NA	3 (NA)	Recurrent GBM	NA	NA	Tumor resection	IL13Ra2	Intracranial	9.6 × 10^8^– 10.6 × 10^8^
Brown et al. (9)	I	1 (100)	Recurrent GBM	Surgery, CCRT with TMZ	MGMT (-) wild IDH (+)	Tumor resection	IL13Ra2	Intracranial	2 × 10^6^–10 × 10^6^
Ahmed et al. (14)	I	17 (47)	Recurrent GBM	Surgery, CCRT with TMZ	NA	None	HER2	Intravenous	1 × 10^6^–1 × 10^8^
O’Rourke et al. (15)	I	10 (50)	Recurrent GBM	Surgery, CCRT with TMZ	MGMT (-)	None	EGFRvIII	Intravenous	1 × 10^8^–5 × 10^8^
Goff et al. (8)	I	18 (83)	Recurrent GBM	Surgery, CCRT with TMZ	4 MGMT (+) 14 MGMT (-)	Lymphodepleting chemotherapy	EGFRvIII	Intravenous	1 × 10^7^–6 × 10^10^
Wang et al. (16)	NA	10 (50)	Recurrent GBM	Surgery, CCRT with TMZ	NA	None	EGFRvIII	NA	NA
Durgin et al. (21)	NA	1 (0)	Recurrent GBM	Surgery, CCRT	MGMT (+), wild IDH (+)	None	EGFRvIII	Intravenous	9.2 × 10^7^
Lin et al. (17)	I	3 (67)	Recurrent GBM	Surgery, CCRT with TMZ	NA	Lymphodepleting chemotherapy	EphA2	Intravenous	1 ×10^6^ (cells/kg)

CCRT, concurrent chemoradiotherapy; GBM, glioblastoma multiforme; IDH, isocitrate dehydrogenase; MGMT, methylated O^6^-methylguanine-DNA methyltransferase; NA, not available; TMZ, temozolomide.

#### CAR T Therapy

The details of CAR T-cell therapy are shown in **Table 1**. The targets of CAR T-cell therapy were epidermal growth factor receptor (EGFR) vIII in four studies with 39 patients (8, 15, 16, 21), interleukin (IL) 13Ra2 in two studies with four patients (9, 13), human epidermal growth factor receptor (HER) 2 in one study with 17 patients (14), and erythropoietin-producing human hepatocellular carcinoma (Eph) A2 in one study with three patients. Six studies with 59 patients (8, 14, 15, 17, 21) used intravenous peripheral injection of CAR T-cells targeting HER2, EGFRvIII, or EphA2, whereas two studies with four patients (9, 13) used intracranial injection targeting IL13Ra2. The cumulative dose of T-cells administered ranged from 1 × 10^6^ to 6.3 × 10^10^ (intravenous, 1 × 10^6^–6.3 × 10^10^; intracranial, 5.2 × 10^7^–10.6 × 10^8^). Lymphodepleting chemotherapy was used as pre-treatment in two studies (8, 17), and tumors were resected before administration of CAR T-cells in two studies (9, 13).

#### Treatment Response

**Table 2** summarizes the treatment response and adverse reactions following CAR T-cell therapy for glioblastoma. The response to CAR T-cell therapy was evaluated in 56 patients. The median interval between the initiation of CAR T-cell therapy and assessment was 2.9 months (range, 1–7.5 months) (8, 9, 13–17). Four studies used Response Assessment in Neuro-Oncology (9, 15, 16), one study used Response Evaluation Criteria in Solid Tumors 1.1 (14), and one study used criteria suggested by the Neuro-oncology Working Group guidelines (8). One study did not explicitly mention the method used (13).

**Table 2:** Treatment results following chimeric antigen receptor-T-cell therapy.

Study	Treatment response		Assessment criteria	Assessment time, months	OS after treatment, months	T-cell persistence	MTD,/m^2^	No. of CRS(max.grade)	No. of neurological events (max. grade)
	No. of patients	Best overall response							
Brown et al. (13)	3	CR:2 PD:1	NA	2.0, 2.8, 4.3	Median 10.3 (8.6–13.9)	NA	1x10^8^	0	2 (G3)
Brown et al. (9)	1	CR:1	RANO	7.5	9.8	> 7 days	NA	1 (G2)	1 (G2)
Ahmed et al. (14)	16	PR:1 SD:7 PD:8	RECIST 1.1	1.5	Median 11.1 (4.1–27.2)	> 1 year	1x10^8^	0	2 (G2)
O’Rourke et al. (15)	6	SD:5 PD:1	RANO	1	Median 8.3 (3.3 – 14.8)	> 1 month	1x10^8^	0	1 (G3)
Goff et al. (8)	17	PD:17	Neuro-oncology Working Group guidelines	3	Median 6.9 (2.8–10)*	> 3 months	1x10^10^	2 (G4)	10 (G2)
Wang et al. (16)	10	SD:3 PD:7 (PsP:1)	RANO	3	Median 8.1 (3.4–9.5)	NA	NA	NA	NA
Durgin et al. (21)	1	NA	NA	NA	34	> 29 months	NA	1 (G2)	NA
Lin et al. (17)	3	SD: 1 PD: 2	iRANO	1.4, 2.8	Median 5.4 (2.8-5.9)	> 4 weeks	NA	2 (G2)	0

*Interquartile range.

The numbers in parentheses are 95% confidence intervals unless otherwise indicated.

CR, complete response; CRS, cytokine release syndrome; G, grade; iRANO, immunotherapy response assessment in neuro-oncology; MTD, maximum tolerated dose; NA, not available; OS, overall survival; PD, progressive disease; PR, partial response; PsP, pseudoprogression; RANO, response assessment in neuro-oncology; RECIST, the response evaluation criteria in solid tumors; SD, stable disease.

Two studies focused on tumors treated with CAR T-cells and did not evaluate the new tumor recurrence or untreated tumors at different sites following CAR T-cell therapy (9, 13). Progressive disease (PD), stable disease (SD), partial response (PR), and complete response (CR) were reported in 36 (67.9%), 16 (28.6%), 1 (1.8%), and 3 (5.4%) patients, respectively. CR was achieved only in patients who underwent intracranial and/or intraventricular injection of CAR T-cells targeting IL13Ra2 (9, 13).

#### Adverse Effects

The maximum tolerated dose, which was reported in four studies (8, 13–15), was 1 × 10^8^/m^2^ or 1 × 10^10^/m^2^. Of 63 patients, six (9.5%) developed CRS (grade 2, n = 4; grade 4, n = 1, not explicitly stated, n = 1) and 16 (25.4%) experienced neurological events (due to either CRES or the disease itself) (grade 2, n = 13; grade 3, n = 3). One patient died of significant pulmonary edema, and the remaining patients with adverse events were successfully managed.

#### Meta-Analysis

**Figure 2:**
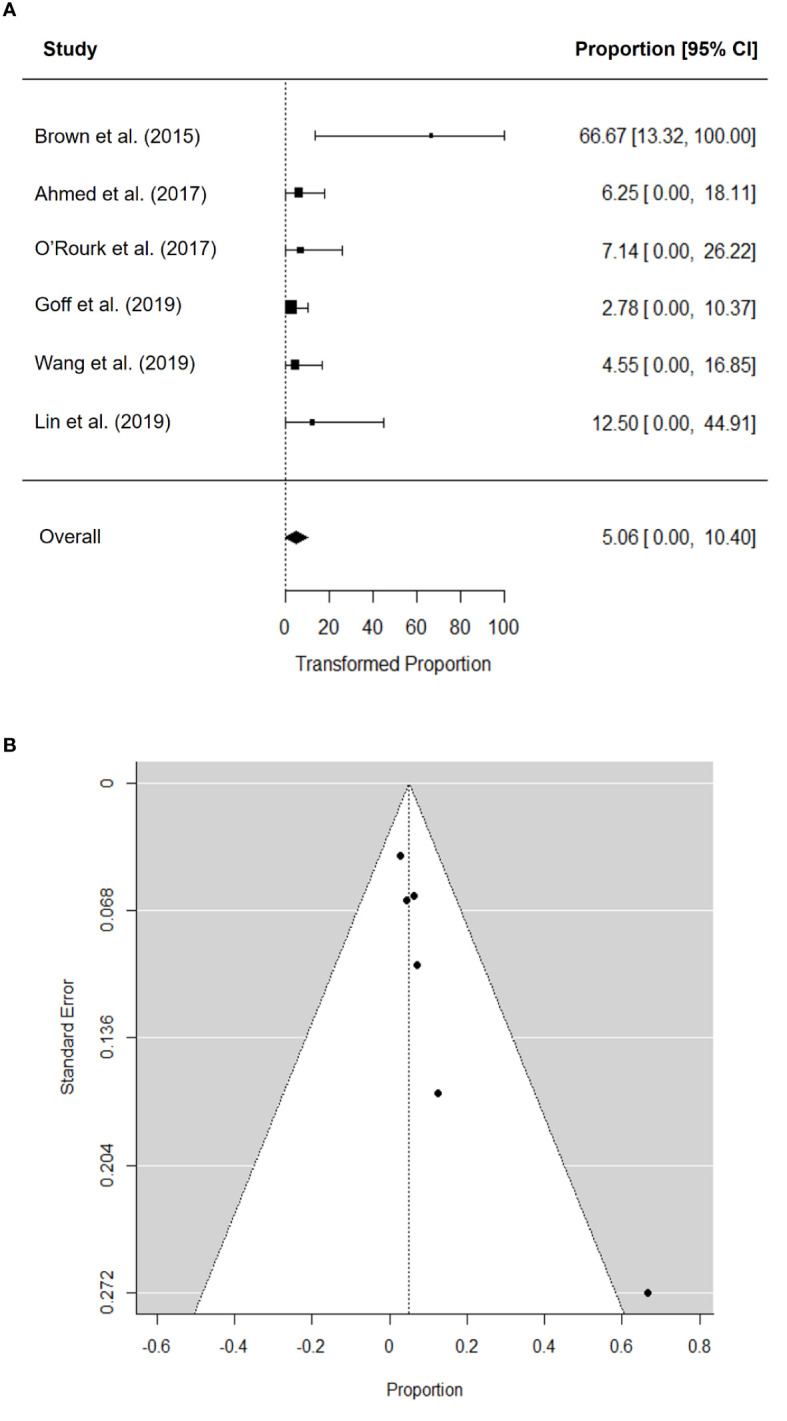
Forest plot for objective response rate **(A)** and funnel plot of publication bias in objective response rate **(B)**.

The pooled ORR was 5.1% (95% confidence interval [CI], 0.0–10.4) (**Figure 2A**). There was no significant heterogeneity in the pooled ORR (*I*^2^ = 0.05%; *P* = 0.329). Visual inspection of the funnel plot (**Figure 2B**) revealed asymmetry, and Begg’s test detected significant publication bias (*P* = 0.017). After performing trim-and-fill adjustment, the adjusted pooled ORR was 3.95% (95% CI, 0.0–9.0).

**Figure 3:**
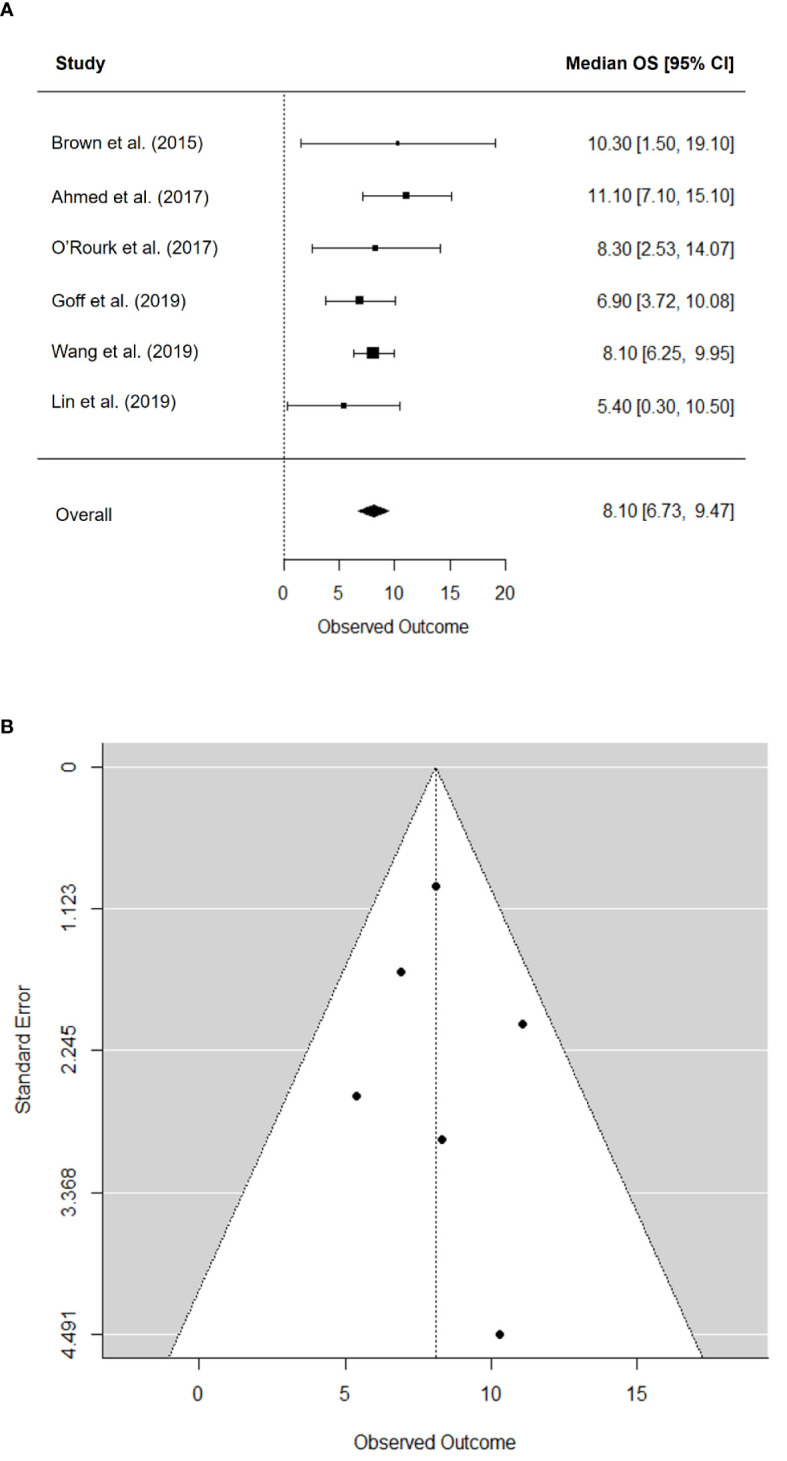
Forest plot for median overall survival **(A)** and funnel plot of publication bias in median overall survival **(B)**.

The pooled median OS was 8.1 months (95% CI, 6.7–9.5) (**Figure 3A**). There was no significant heterogeneity in the pooled median OS (*I^2^* = 0.00%; *P* = 0.545) and no publication bias according to the funnel plot (**Figure 3B**) and Begg’s test (*P* = 0.719).

#### Qualitative Review of Current Issues

The perspectives on the five challenging issues in CAR T-cell therapy in patients with glioblastoma derived from the included studies are summarized in **Table 3**. Six studies (8, 9, 13–15, 17) reported that the generation of CAR technology, CAR T-cells with different targets, transfection methods, and co-stimulators were associated with an acceptable safety profile, whereas T-cell persistence varied. Seven studies with data on the clinical outcome of CAR T-cell therapy (8, 9, 13–17) reported that most patients who underwent intravenous infusion showed SD or PD, whereas CR was only achieved in patients who received locoregional administration of CAR T-cells targeting IL13Ra2. For CRS or CRES, the results of seven studies (8, 9, 13–15, 17) indicated that CRS is not a common or critical issue (9.5%, 6/63) in glioblastoma patients receiving CAR T-cell therapy. Neurological symptoms may occasionally develop during CAR T-cell therapy (25.4%, 16/64), although whether these are attributable to CAR T-cell therapy or related to the tumor itself remains unclear. Histopathological examination of surgical specimens and analysis of serum following CAR T-cell therapy from three studies (13, 15, 21) indicated that antigen loss or downregulation of tumor antigens and increased expression of immunosuppressive molecules are the main causes of recurrence or progression. Differentiating inflammatory changes related to CAR T-cell therapy from tumor progression was difficult by conventional MRI, which showed an increased extent of enhancement or FLAIR signal changes in four studies (13, 15, 16, 21). Evaluation of cerebral blood flow or MR spectroscopy might be helpful to differentiate tumor progression from inflammatory changes (13, 16, 21).

**Table 3:** Challenging issues and current evidence from the included studies.

Source	Methods	Results	Perspective
**I-1. Is CAR technology associated with safety and persistence?**			
Brown et al. (13)	Origin: Autologous Transfection method: Lentiviral Target: IL13Ra2 Co-stimulators: CD3, CD8, CD45RO, CD69, CD95, NKG2d, and CD 28	Safety: Well tolerated and exhibited an acceptable safety profile with limited transient adverse events. Persistence: Very low level of CAR T-cells in the resected tumor after 14 weeks of infusion.	CAR T-cell therapy generated by different T-cell designs is relatively safe. Persistency of CAR T-cells is variable without a noticeable trend according to the design.
Brown et al. (9)	Origin: Autologous, specific for cytomegalovirus Transfection method: Lentiviral Target: IL13Ra2 Co-stimulators: 4-1BB and CD19	Safety: T-cell infusions were not associated with any toxic effects grade 3 or higher. Persistence: CAR T-cells persisted in the cerebrospinal fluid for at least 7 days following intraventricular infusions.	
Ahmed et al. (14)	Origin: Autologous Transfection method: Retroviral Target: Her2 consisting of a murine scFv FRP5 Co-stimulators: CD28	Safety: No dose-limiting toxicity was observed. Two patients (11.8%) had grade 2 seizures and/or headaches, which were probably related to the T-cell infusion. Persistence: Fifteen (88.2%) of 17 patients had the highest frequency of T-cells 3 hours after the infusion and could persist for 1 year at a low frequency.	
O’Rourke et al. (15)	Origin: Autologous Transfection method: Lentiviral Target: EGFRvIII Co-stimulators: CD8, 4-1BB, and CD3ζ signaling	Safety: Three (30%) subjects experienced clinically significant neurologic events, which are common in this population because of the nature of the disease but could also be related to CAR T-cell induced immune responses. Persistence: All subjects had detectable circulating CART-EGFRvIII cells in the first month after infusion. The peak expansion occurred between days 3 and 10 in all subjects.	
Goff et al. (8)	Origin: Autologous Transfection method: Retroviral Target: EGFRvIII Co-stimulators: CD28, 4-1BB, and CD3ζ signaling	Safety: No dose-limiting toxicities associated with cell infusion. Two patients (11.1%) experienced severe hypoxia, including one treatment-related mortality at the highest dose level. Grade 2 neurological symptoms or seizure were observed in 10 patients (55.6%). Persistence: T-cells at 1 month were detectable in 14 (82.4%) of 17 patients.	
Lin et al. (17)	Origin: Autologous Transfection method: Lentiviral Target: EphA2 Co-stimulators: CD8, 4-1BB, and CD3ζ signaling	Safety: Not explicitly mentioned. Persistence: In peripheral blood, CAR T-cells persisted for at least 28 days.	
**I-2. Is locoregional administration of CAR T-cells associated with clinical outcome?**			
Brown et al. (13)	Intracranial administration into the resection cavity *via* the indwelling catheter.	Two CRs (67%) in the resected and injected tumor. However, non-resected and non-injected tumor continued to progress.	Locoregional administration such as intracranial and/or intraventricular could facilitate trafficking and might be associated with better clinical outcomes.
Brown et al. (9)	Intracranial administration into the resection cavity and ventricle *via* the indwelling catheter for brain and leptomeningeal GBMs.	One CR (100%) in the resected and injected tumor. However, the non-resected and non-injected tumor continued to progress and new metastatic lesions developed in the spine. All lesions were indistinct on MRI (i.e., CR) after intraventricular infusion.	
**I-3. Is CRS or CRES significant in the treatment of CAR T for glioblastoma?**			
Brown et al. (13)	Evaluated based on the National Cancer Institute Common Toxicity Criteria version 2.0 after infusion.	Two cases (66.7%) of grade 3 headache in one subject, which were possibly attributable to T-cell administration. Only one (33.3%) case with a grade 3 neurologic event, which included shuffling gait and tongue deviation, possibly attributable to T-cell administration.	Unlike CAR T-cell therapy in hematological malignancy, CRS is not commonly observed in GBM. Mild to moderate neurological events are observed during CAR T-cell therapy, although whether they are attributed to CAR T-cell therapy or related to the tumor itself remains unclear.
Brown et al. (9)	Evaluated based on National Cancer Institute Common Toxicity Criteria for Adverse Events version 4.03. after infusion.	Grade 1 or 2 events that were at least possibly attributable to therapy were observed within 72 hours after the T-cell infusions. These events included headaches, generalized fatigue, myalgia, and olfactory auras, and mostly subsided within 2 days.	
Ahmed et al. (14)	Monitored using the National Cancer Institute Common Terminology Criteria for Adverse Event after infusion.	Two patients (11.8%) had grade 2 seizures and/or headaches, which were probably related to the T-cell infusion.	
O’Rourke et al. (15)	Determined according to National Cancer Institute’s Common Terminology Criteria for Adverse Events version 4.0. after infusion.	None of the patients experienced EGFR-directed toxicity or systemic cytokine release syndrome. Three (30%) subjects experienced clinically significant neurologic events, which are common in this population because of the nature of the disease but could also be related to CAR T EGFR vIII-induced immune responses in the confined intracranial space.	
Goff et al. (8)	NA	No patients required high-dose steroids to ameliorate symptoms of cytokine release syndrome.	
Durgin et al. (21)	NA	Severe headaches requiring the re-initiation of dexamethasone.	
Lin et al. (17)	Monitored according to the National Cancer Institute Common Terminology Criteria for Adverse Events version 4.X.	Two patients (66.7%) experienced grade 2 CRS and returned to normal within 3 weeks after use of dexamethasone. There was no obvious other organ cytotoxicity.	
**I-4. What is the common mechanism of recurrence or progression after CAR T therapy?**			
Brown et al. (13)	Evaluation of surgical specimens using flow cytometry and qPCR analysis.	Targeted IL13Rα2-expressing tumor cells and reduced overall IL13Rα2 expression.	Antigen loss or downregulation of tumor antigens and increased expression of immunosuppressive molecules may be the main cause of recurrence.
O’Rourke et al. (15)	Evaluation of blood samples and surgical specimens using qPCR and RNAscope ISH.	Decreased levels of EGFRvIII and increased expression of immunosuppressive molecules such as IDO1, PD-L1, IL-10, and FoxP3.	
Durgin et al. (21)	Evaluation of surgical specimens using immunohistochemistry and RNA sequencing.	Decreased levels of EGFRvIII and increased expression of immunosuppressive molecules such as PD-L1 and FoxP3.	
**I-5. Can conventional brain MRI findings evaluate the treatment response?**			
Brown et al. (13)	The volume of the region showing contrast enhancement and necrotic tumor tissue were evaluated on FLAIR and CE T1 images. Single voxel MR spectroscopy was also used.	FLAIR: increased signal intensity for several months but subsequently decreased. CE T1: increased enhancement for several months but subsequently decreased. Necrotic tumor volume: increased for several months and persisted. MR spectroscopy: a significant elevation of the lactate and lipid peaks, together with minimal elevation of the choline/creatine ratio.	Conventional MRI might be unreliable for assessing tumor progression post-immunotherapy, as well as the accompanying inflammatory changes (i.e., pseudoprogression). Although they are still investigational, advanced imaging modalities such as perfusion and MR spectroscopy may be valuable in assessing the response to CAR T-cell therapy.
O’Rourke et al. (15)	Evaluated tumor response on FLAIR and CE T1 images, and correlated with surgical specimens.	Increased extent of CE and FLAIR abnormalities, which was interpreted pathologically as favoring treatment effects over true GBM progression in a few patients.	
Wang et al. (16)	Volume, DTI parameters, relative cerebral blood volume, and choline/creatine ratio from enhancing lesions were evaluated and compared.	Six (75%) out of eight lesions demonstrated increased tumor volume, and four (50%) showed decreased relative cerebral flow at follow-up periods relative to baseline. The choline/creatine ratio was slightly decreased compared with that at baseline.	
Durgin et al. (21)	Volume, DTI parameters, relative cerebral blood volume, and choline/creatine ratio from enhancing lesions were evaluated.	A significant reduction in relative cerebral blood volume was strongly associated with tumor proliferative activity.	

CAR, chimeric antigen receptor; CE, contrast-enhanced; CR, complete response; CRES, CAR T-cell related encephalopathy; CRS, cytokine release syndrome; DTI, diffusion tensor imaging; EGFR, epidermal growth factor receptor; Eph, erythropoietin-producing human hepatocellular carcinoma; FLAIR, fluid attenuated inversion recovery; GBM, glioblastoma multiforme; IL, interleukin; ISH, in situ hybridization; MR, magnetic resonance; MRI, magnetic resonance imaging; NK, natural killer; PCR, polymerase chain reaction.

## Discussion

The safety and efficacy of CAR T-cell therapy for glioblastoma are currently under investigation in clinical trials using different cell types based on the encouraging results of CAR T-cell technology in hematologic malignancies. The results of this meta-analysis indicated that CAR T-cell therapy is a relatively safe therapeutic option in glioblastoma within a wide range of cumulative T-cell doses, and that CRS/CRES development is an uncommon and minor issue. However, CAR T-cell therapy is not as effective in glioblastoma as in hematological malignancies, showing a pooled ORR of 5.1% and a pooled median OS of 8.1 months.

Compared with the ORR of 47.5–66.5% and CR rate of 24.4–54.4% in hematological malignancies (6), most glioblastoma patients (67.9%) showed PD after CAR T-cell therapy. This result was in line with those of previous meta-analyses focusing on solid tumors (6, 7). We hypothesized that several factors are associated with the unfavorable response. First, unlike CD19 in hematological malignancies, an ideal target with high homogenous expression and stable expression is absent in glioblastoma. In four studies targeting EGFRvIII, the expression of EGFRvIII was positive in 20–25% of all glioblastomas (22, 23). Although IL13Ra2 is expressed in 80% of glioblastomas and could thus be an ideal target, it is overexpressed in only 44–58% of glioblastomas (24). HER2 is overexpressed in approximately 15% of glioblastomas, which might be a suboptimal target due to lack of high homogeneity (13). Several studies (NCT04385173, NCT04077866, and NCT04045847) are ongoing to identify an optimal antigen such as B7–H3 and CD147. Second, downregulation of the targeted antigen induces an immunosuppressive environment during CAR T-cell therapy (13, 15, 21), and this could underlie progression or recurrence. The use of CAR T-cells targeting multiple tumor-specific antigens may help to overcome this issue. Bi-specific CAR T-cells targeting both HER2 and IL13Rα2 resulted in increased tumor elimination as compared with CAR T-cells targeting a single antigen in a murine model of glioblastoma (25). Tri-specific CAR T-cells targeting HER2, IL13Ra2, and EphA2 achieve a wider coverage of antigens, and studies have shown that this strategy prolongs the survival of mice bearing glioblastoma patient-derived xenografts (26). To overcome the immunosuppressive environment generated during CAR T-cell therapy, combination treatment with immune checkpoint inhibitors could be a promising strategy. Although monotherapy with immune checkpoint inhibitors failed to show a considerable survival benefit in early clinical trials, the addition of PD-1/PD-L1 and CTLA4 inhibitors increases the activity of CAR T-cells in a murine model of glioblastoma (27).

CR was achieved only in patients who underwent intracranial and/or intraventricular administration of CAR T-cells targeting IL13Ra2. There was no CR in patients used intravenous peripheral injection of CAR T-cells targeting other antigens. Glioblastoma was previously believed to break the blood–brain barrier. However, a recent study suggests that the blood–brain barrier cannot be damaged even in the presence of glioblastoma with a significant tumor burden (28). The blood–brain barrier may prevent successful CAR T-cell trafficking to the intracranial tumor after intravenous administration. In a study of 10 glioblastoma patients treated by intravenous administration of EGFRvIII-specific CAR T-cells, heterogeneous CAR T-cell infiltration was observed in the resected tumor specimens from seven patients who had undergone neurosurgical intervention during the course of CAR T-cell therapy (15). Locoregional (intracranial or intraventricular) infusion may be a more effective drug delivery method regarding CAR T-cell trafficking, as demonstrated in a few ongoing clinical trials (NCT04045847 and NCT03283631) targeting two other different antigens (CD147 and EGFRvIII).

Conventional MRI may be insufficient to evaluate the tumor response following CAR T-cell therapy. Although tumors showed an increased extent of signal abnormality and/or enhancement on MRI, tumor volume did not change on follow-up MRI, and pathological examination favored treatment effects over true tumor progression (13, 15). This can be considered as pseudoprogression, which is also observed after chemotherapy or radiation therapy for brain tumors. Perfusion MRI and MR spectroscopy may help to differentiate pseudoprogression from true tumor progression (29). Patients with a favorable response show a significant reduction in relative cerebral blood volume in perfusion MRI and a significant elevation of the lactate and lipid peaks with minimal elevation of the choline/creatine ratio in MR spectroscopy (13, 16, 21).

This study had several limitations. First, large-scale clinical data were lacking, and some included studies showed suboptimal study quality based on the NOS score. Because the application of CAR T-cell therapy in glioblastoma is very recent in the clinical trial setting, few case reports and a limited number of phase I studies with small study populations were available for review. Further reviews including ongoing and upcoming clinical trials are necessary to obtain comprehensive results. Second, significant publication bias was noted in the pooled ORR. We performed trim-and-fill adjustment and the corrected values were presented in this report.

In conclusion, CAR T-cell therapy for glioblastoma is a relatively safe therapeutic option within a wide range of cumulative T-cell doses. CAR T-cell therapy shows marginal efficacy (pooled ORR, 5.1%, and median OS, 8.1 months) and additional investigation is necessary for its application in the treatment of recurrent glioblastoma. Updated results from ongoing and upcoming clinical trials are needed to optimize the application of CAR T-cell therapy in glioblastoma.

## Data Availability Statement

The original contributions presented in the study are included in the article/**Supplementary Material**. Further inquiries can be directed to the corresponding author.

## Author Contributions

JKJ, JP, and KWK contributed to the conception and design of the study. JKJ and JP retrieved studies and data. KWK performed the statistical analysis. JKJ and JP wrote the first draft of the manuscript. CS, HSP, and YKC wrote and revised sections of the manuscript. All authors contributed to manuscript revision, read, and approved the submitted version.

## Funding

This work was supported by the National Research Foundation of Korea (NRF) grant funded by the Korea government(MSIT) (No. NRF-2021R1A2B5B03001891).

## Conflict of Interest

The authors declare that the research was conducted in the absence of any commercial or financial relationships that could be construed as a potential conflict of interest.

## Publisher’s Note

All claims expressed in this article are solely those of the authors and do not necessarily represent those of their affiliated organizations, or those of the publisher, the editors and the reviewers. Any product that may be evaluated in this article, or claim that may be made by its manufacturer, is not guaranteed or endorsed by the publisher.

## References

[1] Ostrom QT Patil N Cioffi G Waite K Kruchko C Barnholtz-Sloan JS. CBTRUS Statistical Report: Primary Brain and Other Central Nervous System Tumors Diagnosed in the United States in 2013-2017. Neuro Oncol (2020) 22:iv1–iv96. doi: 10.1093/neuonc/noaa200 33123732 PMC7596247

[2] Stupp R Taillibert S Kanner A Read W Steinberg D Lhermitte B. Effect of Tumor-Treating Fields Plus Maintenance Temozolomide vs Maintenance Temozolomide Alone on Survival in Patients With Glioblastoma: A Randomized Clinical Trial. JAMA (2017) 318:2306–16. doi: 10.1001/jama.2017.18718 PMC5820703 29260225

[3] Herrlinger U Tzaridis T Mack F Steinbach JP Schlegel U Sabel M. Lomustine-Temozolomide Combination Therapy Versus Standard Temozolomide Therapy in Patients With Newly Diagnosed Glioblastoma With Methylated MGMT Promoter (CeTeG/NOA-09): A Randomised, Open-Label, Phase 3 Trial. Lancet (2019) 393:678–88. doi: 10.1016/s0140-6736(18)31791-4 30782343

[4] Duan J Cui L Zhao X Bai H Cai S Wang G. Use of Immunotherapy With Programmed Cell Death 1 vs Programmed Cell Death Ligand 1 Inhibitors in Patients With Cancer: A Systematic Review and Meta-Analysis. JAMA Oncol (2020) 6:375–84. doi: 10.1001/jamaoncol.2019.5367 PMC6990765 31876895

[5] Conforti F Pala L Bagnardi V De Pas T Martinetti M Viale G. Cancer Immunotherapy Efficacy and Patients' Sex: A Systematic Review and Meta-Analysis. Lancet Oncol (2018) 19:737–46. doi: 10.1016/s1470-2045(18)30261-4 29778737

[6] Grigor EJM Fergusson D Kekre N Montroy J Atkins H Seftel MD. Risks and Benefits of Chimeric Antigen Receptor T-Cell (CAR-T) Therapy in Cancer: A Systematic Review and Meta-Analysis. Transfus Med Rev (2019) 33:98–110. doi: 10.1016/j.tmrv.2019.01.005 30948292

[7] Hou B Tang Y Li W Zeng Q Chang D. Efficiency of CAR-T Therapy for Treatment of Solid Tumor in Clinical Trials: A Meta-Analysis. Dis Markers (2019) 2019:3425291. doi: 10.1155/2019/3425291 30886654 PMC6388318

[8] Goff SL Morgan RA Yang JC Sherry RM Robbins PF Restifo NP. Pilot Trial of Adoptive Transfer of Chimeric Antigen Receptor-Transduced T Cells Targeting EGFRvIII in Patients With Glioblastoma. J Immunother (2019) 42:126–35. doi: 10.1097/cji.0000000000000260 PMC6691897 30882547

[9] Brown CE Alizadeh D Starr R Weng L Wagner JR Naranjo A. Regression of Glioblastoma After Chimeric Antigen Receptor T-Cell Therapy. N Engl J Med (2016) 375:2561–9. doi: 10.1056/NEJMoa1610497 PMC5390684 28029927

[10] Page MJ McKenzie JE Bossuyt PM Boutron I Hoffmann TC Mulrow CD. The PRISMA 2020 Statement: An Updated Guideline for Reporting Systematic Reviews. Bmj (2021) 372:n71. doi: 10.1136/bmj.n71 33782057 PMC8005924

[11] Wells G Shea B O'Connell D Peterson J Welch V Losos M. The Newcastle–Ottawa Scale (NOS) for Assessing the Quality of Non-Randomized Studies in Meta-Analysis (2000). Available at: http://www.ohri.ca/programs/clinical_epidemiology/oxford.asp

[12] Neelapu SS Tummala S Kebriaei P Wierda W Gutierrez C Locke FL. Chimeric Antigen Receptor T-Cell Therapy - Assessment and Management of Toxicities. Nat Rev Clin Oncol (2018) 15:47–62. doi: 10.1038/nrclinonc.2017.148 28925994 PMC6733403

[13] Brown CE Badie B Barish ME Weng L Ostberg JR Chang WC. Bioactivity and Safety of IL13Rα2-Redirected Chimeric Antigen Receptor CD8+ T Cells in Patients With Recurrent Glioblastoma. Clin Cancer Res (2015) 21:4062–72. doi: 10.1158/1078-0432.Ccr-15-0428 PMC4632968 26059190

[14] Ahmed N Brawley V Hegde M Bielamowicz K Kalra M Landi D. HER2-Specific Chimeric Antigen Receptor-Modified Virus-Specific T Cells for Progressive Glioblastoma: A Phase 1 Dose-Escalation Trial. JAMA Oncol (2017) 3:1094–101. doi: 10.1001/jamaoncol.2017.0184 PMC5747970 28426845

[15] O'Rourke DM Nasrallah MP Desai A Melenhorst JJ Mansfield K Morrissette JJD. A Single Dose of Peripherally Infused EGFRvIII-Directed CAR T Cells Mediates Antigen Loss and Induces Adaptive Resistance in Patients With Recurrent Glioblastoma. Sci Transl Med (2017) 9:1–30. doi: 10.1126/scitranslmed.aaa0984 PMC5762203 28724573

[16] Wang S O'Rourke DM Chawla S Verma G Nasrallah MP Morrissette JJD. Multiparametric Magnetic Resonance Imaging in the Assessment of Anti-EGFRvIII Chimeric Antigen Receptor T Cell Therapy in Patients With Recurrent Glioblastoma. Br J Cancer (2019) 120:54–6. doi: 10.1038/s41416-018-0342-0 PMC6325110 30478409

[17] Lin Q Ba T Ho J Chen D Cheng Y Wang L. First-In-Human Trial of EphA2-Redirected CAR T-Cells in Patients With Recurrent Glioblastoma: A Preliminary Report of Three Cases at the Starting Dose. Front Oncol (2021) 11:694941. doi: 10.3389/fonc.2021.694941 34235085 PMC8256846

[18] Viechtbauer W. Conducting Meta-Analyses in R With the Metafor Package. J Stat Software (2010) 36:1 – 48. doi: 10.18637/jss.v036.i03

[19] McGrath S Sohn H Steele R Benedetti A. Meta-Analysis of the Difference of Medians. Biom J (2020) 62:69–98. doi: 10.1002/bimj.201900036 31553488

[20] Balduzzi S Rücker G Schwarzer G. How to Perform a Meta-Analysis With R: A Practical Tutorial. Evid Based Ment Health (2019) 22:153. doi: 10.1136/ebmental-2019-300117 31563865 PMC10231495

[21] Durgin JS Henderson F Jr. Nasrallah MP Mohan S Wang S Lacey SF. Case Report: Prolonged Survival Following EGFRvIII CAR T Cell Treatment for Recurrent Glioblastoma. Front Oncol (2021) 11:669071. doi: 10.3389/fonc.2021.669071 34026647 PMC8138201

[22] Furnari FB Cloughesy TF Cavenee WK Mischel PS. Heterogeneity of Epidermal Growth Factor Receptor Signalling Networks in Glioblastoma. Nat Rev Cancer (2015) 15:302–10. doi: 10.1038/nrc3918 PMC4875778 25855404

[23] Del Vecchio CA Giacomini CP Vogel H Jensen KC Florio T Merlo A. EGFRvIII Gene Rearrangement is an Early Event in Glioblastoma Tumorigenesis and Expression Defines a Hierarchy Modulated by Epigenetic Mechanisms. Oncogene (2013) 32:2670–81. doi: 10.1038/onc.2012.280 22797070

[24] Brown CE Starr R Aguilar B Shami AF Martinez C D'Apuzzo M. Stem-Like Tumor-Initiating Cells Isolated From IL13Rα2 Expressing Gliomas Are Targeted and Killed by IL13-Zetakine-Redirected T Cells. Clin Cancer Res (2012) 18:2199–209. doi: 10.1158/1078-0432.Ccr-11-1669 PMC3578382 22407828

[25] Hegde M Mukherjee M Grada Z Pignata A Landi D Navai SA. Tandem CAR T Cells Targeting HER2 and IL13Rα2 Mitigate Tumor Antigen Escape. J Clin Invest (2016) 126:3036–52. doi: 10.1172/jci83416 PMC4966331 27427982

[26] Bielamowicz K Fousek K Byrd TT Samaha H Mukherjee M Aware N. Trivalent CAR T Cells Overcome Interpatient Antigenic Variability in Glioblastoma. Neuro Oncol (2018) 20:506–18. doi: 10.1093/neuonc/nox182 PMC5909636 29016929

[27] Shen SH Woroniecka K Barbour AB Fecci PE Sanchez-Perez L Sampson JH. CAR T Cells and Checkpoint Inhibition for the Treatment of Glioblastoma. Expert Opin Biol Ther (2020) 20:579–91. doi: 10.1080/14712598.2020.1727436 PMC7202971 32027536

[28] Sarkaria JN Hu LS Parney IF Pafundi DH Brinkmann DH Laack NN. Is the Blood-Brain Barrier Really Disrupted in All Glioblastomas? A Critical Assessment of Existing Clinical Data. Neuro Oncol (2018) 20:184–91. doi: 10.1093/neuonc/nox175 PMC5777482 29016900

[29] Thust SC van den Bent MJ Smits M. Pseudoprogression of Brain Tumors. J Magn Reson Imaging (2018) 48:571–89. doi: 10.1002/jmri.26171 PMC6175399 29734497

