# Supplementary material for: Safety and Efficacy of Chimeric Antigen Receptor T-Cell Therapy for Glioblastoma: A Systemic Review and Meta-Analysis
Source: Front Oncol. 2022 May 26;12:851877. doi: 10.3389/fonc.2022.851877 (PMC9178287; doi:10.3389/fonc.2022.851877)
Supplement: Supplementary file 1 [file Table_1.docx]

**Supplementary Material**

**Supplementary Table 1. Search terms for MEDLINE, EMBASE, and Cochrane Library**

| **Search** | **Query for MEDLINE** |
| --- | --- |
| #1 | "Receptors, Chimeric Antigen"[Mesh] |
| #2 | Chimeric antigen[TW] AND receptor*[TW] |
| #3 | CAR-T[TW] |
| #4 | (modified[TW] OR engineered[TW]) AND T-cell*[TW] |
| #5 | #1 OR #2 OR #3 OR #4 |
| #6 | "Immunotherapy, Adoptive"[Mesh] |
| #7 | therap*[TW] OR treat*[TW] OR immunity[TW] OR immunotherap*[TW] |
| #8 | #6 OR #7 |
| #9 | "Brain Neoplasms"[Mesh] |
| #10 | (Brain*[TW] OR cerebr*[TW] OR Intracranial*[TW]) AND (Neoplasm*[TW] OR Neoplasia*[TW] OR cancer[TW] OR cancers[TW] OR malignan*[TW] OR tumor*[TW] OR tumour*[TW] OR tumuor*[TW] OR carcinoma*[TW] OR adenocarcinoma*[TW] OR adeno-carcinoma*[TW] OR Metast*[TW]) |
| #11 | "Glioblastoma"[Mesh] OR "Astrocytoma"[Mesh:NoExp] |
| #12 | Glioblastoma*[TW] OR Astrocytoma*[TW] OR gliobastoma*[TW] OR glyoblastoma*[TW] OR GBM[TW] OR "malignant glioma*"[TW] |
| #13 | #9 OR #10 OR #11 OR #12 |
| #14 | #5 AND #8 AND #13 |
| #15 | #14 NOT (Autobiography[ptyp] OR Bibliography[ptyp] OR Biography[ptyp] OR pubmed books[filter] OR Comment[ptyp] OR Dataset[ptyp] OR Dictionary[ptyp] OR Editorial[ptyp] OR Electronic Supplementary Materials[ptyp] OR Interview[ptyp] OR Legislation[ptyp] OR News[ptyp] OR Newspaper Article[ptyp] OR Retracted Publication[sb] OR Retraction of Publication[sb] OR Technical Report[ptyp] OR Letter[ptyp]) |
| #16 | #15 AND English[Lang] NOT (animals[Mesh:noexp] NOT (animals[Mesh:noexp] AND humans[Mesh])) |
| **Search** | **Query for EMBASE** |
| #1 | 'chimeric antigen receptor T-cell immunotherapy'/exp |
| #2 | (Chimeric antigen NEAR/3 receptor* NEAR/6 (therap* OR treat* OR immunity OR immunotherap*)):ab,ti,kw |
| #3 | (CAR NEAR/3 T NEAR/6 (therap* OR treat* OR immunity OR immunotherap*)):ab,ti,kw |
| #4 | ((modified OR engineered) NEAR/3 T-cell*):ab,ti,kw |
| #5 | #1 OR #2 OR #3 OR #4 |
| #6 | 'brain cancer'/exp |
| #7 | ((Brain* OR cerebr* OR Intracranial*) NEAR/6 (Neoplasm* OR Neoplasia* OR cancer OR cancers OR malignan* OR tumor* OR tumour* OR tumuor* OR carcinoma* OR adenocarcinoma* OR adeno-carcinoma* OR Metast*)):ab,ti,kw |
| #8 | 'glioblastoma'/exp OR 'astrocytoma'/de |
| #9 | (Glioblastoma* OR Astrocytoma* OR gliobastoma* OR glyoblastoma* OR GBM OR 'malignant glioma*'):ab,ti,kw |
| #10 | #6 OR #7 OR #8 OR #9 |
| #11 | #5 AND #10 |
| #12 | #11 AND ([english]/lim) NOT ('animal'/de NOT ('animal'/de AND 'human'/exp)) |
| #13 | #12 AND ([article]/lim OR [article in press]/lim OR [review]/lim) |
| **Search** | **Query for Cochran Library** |
| #1 | [mh "Receptors, Chimeric Antigen"] |
| #2 | ((Chimeric antigen NEAR/3 receptor*):ab,ti,kw |
| #3 | CAR-T |
| #4 | (modified OR engineered) NEAR/3 "T cell*" |
| #5 | #1 OR #2 OR #3 OR #4 |
| #6 | [mh "Immunotherapy, Adoptive"] |
| #7 | therap* OR treat* OR immunity OR immunotherap* |
| #8 | #6 OR #7 |
| #9 | [mh "Brain Neoplasms"] |
| #10 | (Brain* OR cerebr* OR Intracranial*) NEAR/6 (Neoplasm* OR Neoplasia* OR cancer OR cancers OR malignan* OR tumor* OR tumour* OR tumuor* OR carcinoma* OR adenocarcinoma* OR adeno-carcinoma* OR Metast*) |
| #11 | [mh "Glioblastoma"] OR [mh ^"Astrocytoma"] |
| #12 | Glioblastoma* OR Astrocytoma* OR gliobastoma* OR glyoblastoma* OR GBM OR "malignant glioma*" |
| #13 | #9 OR #10 OR #11 OR #12 |
| #14 | #5 AND #8 AND #13 |
| #15 | #14 in Reviews, Trials(Published) |

| **Study** | **Selection of cohorts** | | | | **Comparability of cohorts** | **Outcome** | | |
| --- | --- | --- | --- | --- | --- | --- | --- | --- |
|  | Representativeness of the exposed cohort | Selection  of the nonexposed  cohort | Ascertainment of  exposure | Demonstration that  outcome of interest  was not present at  start of study ^a^ | Comparability of cohorts on  the basis of the design  or analysis | Ascertainment of  Outcome | Adequate  follow-up ^b^ | Adequacy  of follow-up of  cohorts ^c^ |
| Brown et al. (2015) |  |  | * | * |  | * | * | * |
| Brown et al. (2016) |  |  | * | * |  | * | * | * |
| Ahmed et al. (2017) |  |  | * | * |  | * | * | * |
| O'Rourke et al. (2017) |  |  | * | * |  | * | * |  |
| Goff et al. (2019) |  |  | * | * |  | * | * | * |
| Wang et al. (2019) |  | * | * | * |  | * | * | * |
| Durgin et al. (2021) |  |  | * | * |  |  | * | * |
| Lin et al.  (2021) |  |  | * | * |  | * | * | * |

**Supplementary Table 2. The Newcastle-Ottawa scale (NOS) quality assessment of the enrolled studies**

Each study could be awarded a maximum of nine stars: a maximum of two stars for the item regarding comparability and a maximum of one star for the other seven items.

^a^One star was awarded if a study was a prospective cohort study.

^b^All studies were awarded if the study reported overall survival and/or clinical response along with the time.

^c^If a study reported a follow up rate of ≥ 80%, one star was awarded.
